# Supplementary material for: Social Vulnerability of Brazilian Metropolitan Schools and Teachers’ Absence from Work Due to Vocal and Psychological Symptoms: A Multilevel Analysis
Source: Int J Environ Res Public Health. 2023 Feb 8;20(4):2972. doi: 10.3390/ijerph20042972 (PMC9966546; doi:10.3390/ijerph20042972)
Supplement: Supplementary file 1 [file ijerph-20-02972-s001.zip › ijerph-2150096-supplementary.pdf]

Social vulnerability of Brazilian metropolitan schools and teachers' absence from work due to vocal and psychological symptoms: a multilevel analysis

Supplementary Material

Extended Methods

Let Y_{ij} be 1 if the ith teacher at the jth FU missed school due to a voice/psychological symptoms and zero otherwise, then the log odds of the ith teacher at the jth FU missing school was modeled as follows:

Y_{ij} ~Bernoulli($P(Y_{ij}=1)$)

$$\text{Logit}[P(Y_{ij}=1 \mid \beta, u_{0j})] = \beta_0 + \beta_1\text{Sex} + \beta_2\text{Age}_{(35\text{-}44)} + \beta_3\text{Age}_{(45\text{-}54)} + \beta_4\text{Age}_{(\geq 55)} + \beta_5\text{SVI}_{(\text{Median})} + \\ + \beta_6\text{SVI}_{(\text{High})} + \ldots + u_{0j}$$

Where,

Sex =1 if Woman; 0 otherwise

$\text{Age}_{(35\text{-}44)}$=1 if 35-44 years old; 0 otherwise

$\text{Age}_{(55\text{-}54)}$=1 if 45-54 years old; 0 otherwise

$\text{Age}_{(\geq 55)}$=1 if ≥55 years old; 0 otherwise

$\text{SVI}_{(\text{Median})}$=1 if Median SVI median level; 0 otherwise

$\text{SVI}_{(\text{High})}$=1 if Median SVI high level; 0 otherwise

β_0 – is the intercept, represents the log odds of a teacher missing school in an average FU;

u_{0j} – is the random intercept for FU, describes the variation of the jth FU intercept relative to the average FU, $u_{0j} \sim N(0,\sigma_{u0})$;

At an average FU (i.e. a FU whose random effect is equal to zero on the logit scale), the probability of Y=1 is $\frac{\exp(\beta_0+\beta 1\ \text{Sex}+\beta 2\ \text{Age}(35-44)\ +\ \beta 3\text{Age}(45-54)\ +\ \beta 4\ \text{Age}(\geq 55)\ +\ \beta 5\ \text{SVI(Median)}\ +\ \beta 6\ \text{SVI(High)}\ +\ \ldots)}{1+\exp(\beta_0\beta 1\ \text{Sex}+\beta 2\ \text{Age}(35-44)\ +\ \beta 3\text{Age}(45-54)\ +\ \beta 4\ \text{Age}(\geq 55)\ +\ \beta 5\ \text{SVI(Median)}\ +\ \beta 6\ \text{SVI(High)}\ +\ \ldots)}$.

$\beta_{1...}$ – are the fixed effects of teacher's demographic characteristics, working conditions and the SVI, conditional on the FU intercept and other covariates, represent log odds of missing school when comparing two teachers , one presenting that characteristic relative to the reference level of the characteristic, but who share identical values on the remaining covariates and who also share the same FU average risk (i.e., the value of the random effect).

… represents all other fixed effects listed in the table below.

**Covariate**	Categories
**Sex**	Male
	Female
**Age (years)**	≤34
	35-44
	45-54
	≥55
**Working weeks longer than 40 hours**	No
	Yes
**Employment time (years)**	<10
	10-20
	>20
**Income from school in minimum wages**	> three
	Up to three
**Working in more than one school**	No
	Yes
**Working in another activity**	No
	Yes
**School administration**	Private
	Municipal
	State
	Federal
**School size by the number of teachers**	≤10
	10 - 20
	21-30
	>30
**Perception of intense noise at school**	Never or almost never /Rarely
	Often/Sometimes
**Student indiscipline**	Never or almost never /Rarely
	Often/Sometimes
**Roundtrip home-work commute**	10 - 20
	21-50

**(in minutes)**	>51
**Experiencing verbal violence in school**	Never
	Once/twice or more
**Experiencing physical violence in school**	Never
	Once/twice or more
**Excessive workload**	Never or almost never /Rarely
	Often/Sometimes
**Few opportunities to learn new things**	Never or almost never /Rarely
	Often/Sometimes
**Insufficient time to complete tasks**	Never or almost never /Rarely
	Often/Sometimes
**Limited autonomy**	Never or almost never /Rarely
	Often/Sometimes
**Low social support**	Never or almost never /Rarely
	Often/Sometimes
**SVI of the municipalities**	Low
	Median
	High
